# Efficacy of Large Language Models in Providing Evidence-Based Patient Education for Celiac Disease: A Comparative Analysis

**DOI:** 10.3390/nu17243828

**Published:** 2025-12-06

**Authors:** Luisa Bertin, Federica Branchi, Carolina Ciacci, Anne R. Lee, David S. Sanders, Nick Trott, Fabiana Zingone

**Affiliations:** 1 Department of Surgery, Oncology, Gastroenterology, University of Padua, 35128 Padua, Italy; 2 Gastroenterology Unit, Azienda Ospedale-Università Padova, 35128 Padua, Italy; 3 Medizinische Klinik Für Gastroenterologie, Infektiologie und Rheumatologie, Charité—Universitätsmedizin Berlin, 12203 Berlin, Germany; 4 Department of Medicine, Surgery and Dentistry, Scuola Medica Salernitana, University of Salerno, 84081 Salerno, Italy; 5 Department of Medicine, Celiac Disease Center, Columbia University Medical Center, Columbia University, New York, NY 10032, USA; 6 Gastroenterology and Liver Unit, Royal Hallamshire Hospital, Sheffield S5 7AU, UK

**Keywords:** artificial intelligence, patient education, gluten, health literacy

## Abstract

**Background/Objectives**: Large language models (LLMs) show promise for patient education, yet their safety and efficacy for chronic diseases requiring lifelong management remain unclear. This study presents the first comprehensive comparative evaluation of three leading LLMs for celiac disease patient education. **Methods**: We conducted a cross-sectional evaluation comparing ChatGPT-4, Claude 3.7, and Gemini 2.0 using six blinded clinical specialists (four gastroenterologists and two dietitians). Twenty questions spanning four domains (general understanding, symptoms/diagnosis, diet/nutrition, lifestyle management) were evaluated for scientific accuracy, clarity (5-point Likert scales), misinformation presence, and readability using validated computational metrics (Flesch Reading Ease, Flesch-Kincaid Grade Level, SMOG index). **Results**: Gemini 2.0 demonstrated superior performance across multiple dimensions. Gemini 2.0 achieved the highest scientific accuracy ratings (median 4.5 [IQR: 4.5–5.0] vs. 4.0 [IQR: 4.0–4.5] for both competitors, *p* = 0.015) and clarity scores (median 5.0 [IQR: 4.5–5.0] vs. 4.0 [IQR: 4.0–4.5], *p* = 0.011). While Gemini 2.0 showed numerically lower misinformation rates (13.3% vs. 23.3% for ChatGPT–4 and 24.2% for Claude 3.7), differences were not statistically significant (*p* = 0.778). Gemini 2.0 achieved significantly superior readability, requiring approximately 2–3 fewer years of education for comprehension (median Flesch-Kincaid Grade Level 9.8 [IQR: 8.8–10.3] vs. 12.5 for both competitors, *p* < 0.001). However, all models exceeded recommended 6th–8th grade health literacy targets. **Conclusions**: While Gemini 2.0 demonstrated statistically significant advantages in accuracy, clarity, and readability, misinformation rates of 13.3–24.2% across all models represent concerning risk levels for direct patient applications. AI offers valuable educational support but requires healthcare provider supervision until misinformation rates improve.

## 1. Introduction

The emergence of sophisticated large language models (LLMs) represents a transformative advancement in healthcare communication and patient education that directly addresses critical gaps in chronic disease management [1,2,3,4,5,6,7,8,9,10]. While these Artificial Intelligence (AI) technologies demonstrate remarkable capabilities in processing complex medical information and generating human-like text, their practical utility and safety in delivering patient-oriented medical education remain subjects of critical evaluation, particularly in conditions requiring lifelong lifestyle modifications [11,12,13,14,15,16,17,18,19,20,21].

Celiac disease is an immune-mediated enteropathy triggered by gluten ingestion in genetically predisposed individuals and affects approximately 1% of the population globally, with most cases remaining undiagnosed due to varied clinical presentations and limited awareness [22,23,24,25,26,27,28,29,30,31,32,33,34]. The condition necessitates lifelong adherence to a strict gluten-free diet (GFD) as its only effective treatment [35,36,37,38,39,40,41,42,43,44,45], making comprehensive patient education fundamental to successful disease management [46]. The management of celiac disease presents multifaceted educational challenges that test the capabilities of both traditional education methods and emerging AI technologies [47,48,49,50,51,52,53,54].

Patients require understanding of the disease’s pathophysiology, recognition of symptoms beyond classical gastrointestinal presentations, interpretation of diagnostic testing including serological markers and histological findings [55,56], implementation of a strict gluten-free diet while maintaining nutritional balance, and regular monitoring for complications and response to treatment [57,58,59]. Additionally, patients must manage psychological impacts including anxiety, depression, and reduced quality of life, which have been shown to directly impact dietary adherence [60,61,62,63,64,65,66,67,68,69,70]. Research has identified also other critical factors influencing dietary compliance, including cognitive elements (disease knowledge, label interpretation capabilities, food intolerance awareness), psychological aspects (emotional responses including frustration, mood disorders, stress), and social-cultural considerations (community understanding, dining challenges, travel complications, social gatherings, and financial impacts of specialty foods) [71,72,73,74,75,76,77]. Educational gaps regarding both the condition and gluten-free requirements frequently contribute to poor compliance, with adherence rates ranging from 42–91% in adults [78,79]. Recent surveys of 2437 patients and 346 healthcare professionals exposed concerning knowledge gaps: 20% of patients expressed uncertainty about medications and gluten, nearly one–fifth of healthcare professionals incorrectly believed pseudocereals contain gluten, and 48% of healthcare professionals allocated less than 15 min to explain the gluten-free diet at diagnosis, insufficient time given that 96% acknowledge needing more training in this area [80]. The digital information landscape further emphasizes the need for reliable AI tools, with 51.4% of patients turning to the internet and social media for GFD guidance while only 15.2% consult their physicians for dietary questions. This digital-first behavior pattern, coupled with limited healthcare access to registered dietitian-nutritionists, whom 93.4% of healthcare professionals believe should be more integrated into care teams, creates an urgent need for scalable, accurate educational solutions that AI models could potentially provide. Moreover, Sorin et al.’s systematic review on LLMs and empathy found that ChatGPT’s responses were preferred over physician responses in 78.6% of cases when answering patient questions, and were rated significantly higher for both quality and empathy [81].

Despite the proliferation of studies examining LLM applications in patient education across various medical conditions, a critical gap exists in the literature: no study has systematically compared the performance of multiple leading LLMs specifically for celiac disease education. This gap is particularly consequential given the unique multi-dimensional educational complexity inherent to celiac disease management. Patients require accurate, accessible information spanning pathophysiology, dietary restrictions, cross-contamination prevention strategies, nutritional supplementation, and long-term monitoring protocols. Consequently, clinicians and healthcare organizations currently lack evidence-based guidance for selecting among available LLMs when developing patient education resources for this prevalent chronic condition.

Among the leading LLMs, ChatGPT-4 (OpenAI), Claude 3.7 (Anthropic), and Gemini 2.0 (Google) each demonstrate distinct approaches to medical information processing, empathetic engagement, and clinical accuracy that warrant systematic comparison.

This study makes several novel contributions: (1) the first multi-dimensional comparative framework for evaluating LLMs in celiac disease patient education; (2) a rigorous evaluation methodology integrating multi-expert blinded review with statistical verification across scientific accuracy, clarity, misinformation detection, and validated readability metrics; and (3) inclusion of both gastroenterologists and dietitians, enabling profession-specific perspectives on AI-generated dietary content. These methodological innovations provide a replicable template for future evaluations of AI models in chronic disease education. As digital health tools become increasingly integrated into patient care pathways, this evaluation offers timely insights into how these emerging technologies can potentially address the well-documented educational gaps that contribute to poor GFD adherence and subsequent health outcomes.

## 2. Materials and Methods

### 2.1. Study Design

We conducted a comprehensive cross-sectional evaluation study to assess clinical accuracy, clarity, and potential for misinformation in AI-generated responses to celiac disease-related questions. This study employed a multi-expert evaluation framework with robust statistical analysis to compare three leading LLMs: ChatGPT-4, Claude 3.7, and Google’s Gemini 2.0. The objective was to systematically evaluate their efficacy, accuracy, and comprehensibility in providing patient education information on celiac disease.

### 2.2. Expert Panel Composition and Characteristics

We assembled a multidisciplinary expert panel of six clinical specialists with demonstrated expertise in celiac disease management. All experts were actively engaged in clinical practice involving celiac disease management and had published peer-reviewed research in the field: The expert panel had substantial experience in celiac disease management, with a mean of 19.7 years of specialized experience (SD = 7.6 years; range: 10–30 years), including four gastroenterologists with two specialized dietitians. Expert selection followed purposive sampling to ensure representation across relevant medical specialties while maintaining clinical expertise standards.

To minimize bias, experts were blinded to AI model identities during the evaluation process. Evaluators underwent a 1 h training session to establish consistent rating criteria prior to assessment. Each expert independently evaluated all 60 responses (20 questions × 3 LLMs) in randomized order to minimize sequential and learning biases. All questions and AI-generated responses were presented in English to ensure consistency across all evaluations. While the expert panel included both native English speakers (n = 3) and non-native English speakers (n = 3), all non-native speakers demonstrated high English proficiency (estimated C1/C2 level based on their international publication records and clinical practice in English-speaking or international contexts), minimizing potential language-related bias in clarity assessments.

### 2.3. Question Development and Content Validation

We developed a comprehensive set of 20 clinically relevant questions spanning four critical domains of celiac disease management:

- General understanding (n = 5): Fundamental concepts, etiology, and disease characteristics.
- Symptoms and diagnosis (n = 5): Clinical presentation, diagnostic criteria, and testing protocols.
- Diet and nutrition (n = 5): Dietary management, food safety, and nutritional considerations.
- Lifestyle and management (n = 5): Long-term care, monitoring, and treatment adherence.

Questions were developed through iterative expert consultation and content validation. Initial question pools were reviewed by an independent gastroenterologist not involved in the evaluation process. Content validity was established through expert consensus review, with all questions approved by independent gastroenterology specialists. Questions were designed to reflect common patient inquiries encountered in clinical practice and covered varying complexity levels from basic patient education to nuanced clinical decision-making scenarios. While these 20 questions were designed to represent the core educational needs of celiac disease patients, we acknowledge that this focused set may not capture all possible patient inquiries or the full complexity of celiac disease education. The questions were selected to balance comprehensiveness with practical evaluation constraints, prioritizing domains most frequently encountered in clinical practice.

The questions are reported in Table 1.

### 2.4. AI Model Selection and Configuration

Three state-of-the-art large language models were selected based on their widespread clinical adoption and demonstrated performance in medical domains:

- ChatGPT-4 (OpenAI): GPT-4 architecture accessed via ChatGPT interface.
- Claude 3.7 (Anthropic): Claude-3.7 Sonnet model via Claude interface.
- Gemini 2.0 (Google): Gemini 2.0 model via Google AI interface.

All models were accessed using their standard and free public interfaces with default settings during the data collection period (January 2025–April 2025). No fine-tuning or specialized medical prompting was employed to ensure evaluation reflected real-world clinical usage scenarios. Each question was posed identically across all three models using neutral, clinically relevant phrasing. A total of 360 responses were successfully collected (120 responses per model), ensuring balanced comparison across all AI systems.

### 2.5. Evaluation of Metrics and Rating Scales

#### 2.5.1. Primary Outcome Measures

The following criteria were employed:

1. Scientific accuracy: Rated on a 5-point Likert scale (1 = contains serious scientific inaccuracies; 3 = mostly accurate with minor errors; 5 = completely accurate based on current evidence-based guidelines). Experts were instructed to reference the latest guidelines and consensus recommendations [46,56].
2. Clarity of information: Rated on a 5-point Likert scale (1 = very unclear, confusing presentation; 3 = moderately clear with some ambiguity; 5 = exceptionally clear, well-organized, and comprehensible to patients). Evaluators assessed structural organization, terminology appropriateness, and explanation quality.

#### 2.5.2. Secondary Outcome Measures

The following parameters were evaluated.

1. Presence of misinformation: Binary assessment (Yes/No) with required justification for “Yes” responses. Misinformation was defined as any statement that contradicted established clinical guidelines, contained factual errors that could potentially impact patient care, or presented unsubstantiated or controversial claims as established facts.
2. Readability analysis: Objective linguistic assessment was performed using validated computational readability metrics:

- Flesch Reading Ease: Scale from 0–100 with higher scores indicating easier readability (90–100: very easy; 80–89: easy; 70–79: fairly easy; 60–69: standard; 50–59: fairly difficult; 30–49: difficult; 0–29: very confusing) [82].
- Flesch-Kincaid Grade Level: Estimates the U.S. academic grade level required to comprehend the text [83].
- Simple Measure of Gobbledygook (SMOG) Index: Calculates the years of education needed to understand the text, with particular relevance for healthcare materials [84].

Text preprocessing included removal of formatting elements while preserving punctuation, sentence structure, and paragraph organization.

### 2.6. Expert Assessment Protocol

Experts independently evaluated all AI-generated responses using a standardized web-based assessment platform. The evaluation protocol incorporated several bias-reduction strategies: complete model anonymization with responses labeled only as “Model A,” “Model B,” and “Model C”; response order randomization for each expert to minimize sequence effects; standardized evaluation interfaces and rating scales across all experts; and independent evaluation without inter-expert communication during the assessment period.

### 2.7. Statistical Analysis

Descriptive statistics were calculated for all evaluation metrics, including medians, interquartile ranges, means, and standard deviations. Values were presented as median [IQR] for continuous variables and % (n/N) for categorical variables.

Friedman tests were used to assess overall differences between the three AI models for continuous outcomes (scientific accuracy and clarity ratings), with Kendall’s coefficient of concordance (W) calculated as effect size measures for readability metrics. Post hoc pairwise comparisons employed Wilcoxon signed-rank tests with comprehensive multiple testing correction applied. Cohen’s d effect sizes were calculated for all pairwise comparisons and interpreted as small (d = 0.2), medium (d = 0.5), or large (d = 0.8) according to established conventions.

One-way ANOVA was used to assess performance differences across the four content categories (General Understanding, Diagnosis and Symptoms, Diet and Nutrition, Lifestyle and Management) within each AI model for both scientific accuracy and clarity ratings.

Overall differences in misinformation rates between models were assessed using Friedman tests. Category-based differences in misinformation presence within each AI model were evaluated using one-way ANOVA.

Spearman correlation analysis was performed to examine pattern similarity between models for accuracy, misinformation and clarity ratings, both overall and within specific content categories.

Independent samples *t*-tests were used to compare ratings between gastroenterologists and dietitians for scientific accuracy, clarity, and misinformation detection across all AI models. Effect sizes were calculated using Cohen’s d with 95% confidence intervals.

Three validated computational linguistic measures were calculated on original AI responses (n = 60): Flesch Reading Ease, Flesch-Kincaid Grade Level, and SMOG Index. Between-model differences were assessed using Friedman tests with Kendall’s coefficient of concordance (W) as effect size measures.

Descriptive analysis of follow-up question generation rates was performed across all AI models using frequency counts and percentages.

Intraclass correlation coefficient (ICC) analysis was performed using ICC(2, k) models appropriate for fixed expert panels, treating experts as fixed effects. 95% confidence intervals were calculated for all ICC estimates. Fleiss’ kappa coefficients assessed inter-expert agreement for misinformation identification beyond chance levels, with z-tests for statistical significance. Cronbach’s alpha coefficients evaluated internal consistency of ratings within each model across all evaluations.

Complete case analysis was employed for the full dataset of 360 responses with no missing data. Quality assurance procedures included complete verification of all AI responses, duplicate detection, and data validation.

All analyses were performed using R version 4.3.0 with key packages including: lme4, psych (reliability analysis), irr (inter-rater reliability), and performance (model diagnostics). A *p*-value below 0.05 was deemed statistically significant.

Sample size calculations targeted detection of medium effect sizes (Cohen’s d = 0.5) with 80% power at α = 0.05 for comparing three AI models. Post hoc pairwise comparisons using Wilcoxon signed-rank tests with Bonferroni correction (α = 0.017) required 84 matched pairs to achieve 80% power for medium effect sizes. The study design of 20 questions evaluated by 6 expert raters across 3 AI models yielded 120 matched observations (120 per model, 360 total evaluations), ensuring adequate statistical power.

### 2.8. Ethical Considerations

This study involved evaluation of publicly available AI model outputs without human subject involvement. No patient data or protected health information was utilized. Expert participation was voluntary with informed consent obtained for evaluation participation and potential publication of aggregate results.

## 3. Results

The study achieved complete data collection with 360 AI-generated responses (120 per model) and comprehensive expert evaluations from all six clinical specialists, yielding zero missing data points.

### 3.1. Scientific Accuracy

**Figure 1:**
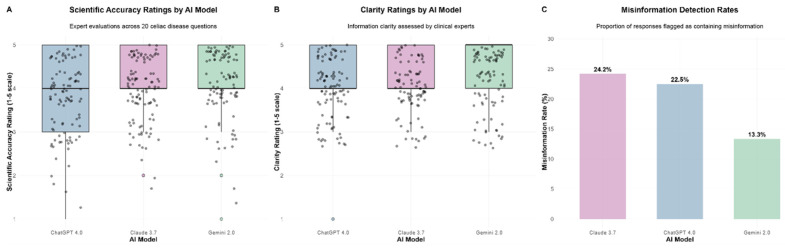
Comparative performance evaluation of three AI models on scientific accuracy, clarity, and misinformation detection. This figure presents a comprehensive evaluation of three AI models (ChatGPT-4, Claude 3.7, and Gemini 2.0) across three key performance metrics. (**A**) Scientific accuracy ratings based on expert evaluations, with individual data points plotted for each model; (**B**) clarity ratings as assessed by clinical experts, using the same evaluation framework; (**C**) misinformation detection rates.

Friedman analysis revealed statistically significant differences in scientific accuracy ratings between AI models (*p* = 0.015). Gemini 2.0 demonstrated superior performance with a median accuracy score of 4.5 [Interquartile Range (IQR): 4.5–5.0], followed by Claude 3.7 and ChatGPT-4 (both median = 4.0 [IQR: 4.0–4.5]). These results are illustrated in Figure 1, together with results on clarity and misinformation detection.

These results are illustrated in Table 2, together with results on clarity, misinformation presence and readability scores.

Post hoc Wilcoxon signed-rank tests with comprehensive multiple testing corrections revealed significant superiority of Gemini 2.0 over ChatGPT-4 (*p* = 0.006, Cohen’s d = −0.70). Claude 3.7 vs. Gemini 2.0 comparison achieved significance (*p* = 0.024), while ChatGPT-4 vs. Claude 3.7 showed no significant difference (*p* = 0.254).

These comparisons are represented in Table 3 along with results on clarity and misinformation, later detailed in the text.

One-way ANOVA revealed no statistically significant differences in scientific accuracy ratings across the four question categories for any AI model. For ChatGPT-4, mean scores ranged from 3.80 (Lifestyle and Management) to 4.30 (General Understanding, *p* = 0.126). Claude 3.7 demonstrated similar patterns with scores ranging from 3.97 to 4.27 (*p* = 0.551). Gemini 2.0 showed the largest numerical differences, with scores ranging from 4.07 (Lifestyle and Management) to 4.53 (General Understanding, *p* = 0.082), approaching but not reaching statistical significance.

### 3.2. Clarity of Information

Clarity ratings exhibited significant between-model variation (*p* = 0.011). Gemini 2.0 achieved the highest clarity ratings with a median of 5.0 [IQR: 4.5–5.0], compared to Claude 3.7 and ChatGPT-4 (both median = 4.0 [IQR: 4.0–4.5], Figure 1 and Table 2).

The most substantial difference was observed between ChatGPT-4 and Gemini 2.0 (*p* = 0.002, Table 3). The ChatGPT-4 vs. Claude 3.7 comparison (*p* = 0.022) achieved statistical significance following multiple testing adjustment, while the Claude 3.7 vs. Gemini 2.0 comparison approached significance (*p* = 0.053).

No significant differences in clarity ratings were observed across question categories for any AI model. ChatGPT-4 scores ranged from 3.93 to 4.27 (*p* = 0.304), Claude 3.7 from 4.23 to 4.33 (*p* = 0.953), and Gemini 2.0 from 4.37 to 4.53 (*p* = 0.823). The narrow range of mean scores across categories suggests consistent performance regardless of topic area.

### 3.3. Misinformation Detection and Assessment

Gemini 2.0 demonstrated the lowest misinformation rate at 13.3% (16/120 evaluations), compared to both ChatGPT-4 (23.3%, 28/120 evaluations) and Claude 3.7 (24.2%, 29/120 evaluations, Figure 1). Overall Friedman test for misinformation rates was non-significant (*p* = 0.778, Table 2). Analysis of misinformation presence showed no significant category-based differences for any AI model (ChatGPT-4, *p* = 0.465; Claude 3.7, *p* = 0.452; Gemini 2.0, *p* = 0.263). Misinformation rates remained consistently low across all categories, with mean scores below 0.35 for all comparisons.

Fleiss’ kappa analysis revealed statistically significant inter-expert agreement for Gemini 2.0 (κ = 0.192, z = 3.33, *p* < 0.001), while agreement for ChatGPT-4 (κ = 0.05, z = 0.86, *p* = 0.389) and Claude 3.7 (κ = 0.10, z = 1.73, *p* = 0.084) remained non-significant.

### 3.4. Correlation Between Accuracy, Clarity and Misinformation

Spearman correlation analysis revealed moderate positive pattern similarity between models for clarity ratings (ChatGPT-4 vs. Claude 3.7: ρ = 0.592, *p* = 0.006), suggesting partially consistent question-difficulty hierarchies. Scientific accuracy correlations were generally weaker, with the strongest association observed between Claude 3.7 and Gemini 2.0 (ρ = 0.461, *p* = 0.041). Category-specific analysis identified significant correlations in Clarity for Diet and Nutrition (Claude 3.7 vs. Gemini 2.0: ρ = 0.90, *p* = 0.037) and Lifestyle and Management (ChatGPT-4 vs. Claude 3.7: ρ = 0.892, *p* = 0.042).

Strong negative correlations emerged between scientific accuracy and misinformation identification across all analyses: overall (ρ = −0.711, *p* < 0.001), ChatGPT-4-specific (ρ = −0.663, *p* = 0.001), Claude 3.7 -specific (ρ = −0.672, *p* = 0.001), and Gemini 2.0-specific (ρ = −0.627, *p* = 0.003). This robust pattern indicates that higher accuracy ratings were consistently associated with lower misinformation detection across all models.

### 3.5. Evaluator Expertise Effects

The comparative analysis of expert evaluations revealed consistent and statistically significant differences between gastroenterologists and dietitians in their assessment of AI-generated responses to diet and nutrition questions related to celiac disease. A total of 30 individual evaluations per AI model were analyzed, comprising 20 assessments from gastroenterologists and 10 assessments from dietitians across five diet and nutrition questions.

For scientific accuracy ratings, gastroenterologists consistently provided higher scores than dietitians across all three AI models. ChatGPT responses received mean scores of 4.2 from gastroenterologists compared to 3.1 from dietitians (*p* = <0.001). Similarly, Claude responses were rated 4.4 by gastroenterologists versus 3.5 by dietitians (*p* = 0.013), while Gemini responses received scores of 4.6 from gastroenterologists compared to 3.4 from dietitians (*p* = 0.007).

Clarity ratings demonstrated a similar pattern of systematic differences between expert groups. Gastroenterologists rated ChatGPT responses significantly higher for clarity (mean = 4.5) compared to dietitians (mean = 3.3, *p*< 0.001). For Claude, gastroenterologists provided clarity scores averaging 4.5 versus 3.8 from dietitians (*p* = 0.019). The largest clarity rating disparity was observed for Gemini, where gastroenterologists averaged 4.8 compared to dietitians’ mean of 3.6 (*p* = 0.0013).

In contrast to accuracy and clarity metrics, misinformation ratings showed no statistically significant differences between expert groups across any AI model. ChatGPT received misinformation scores of 0.15 from gastroenterologists and 0.3 from dietitians (mean difference = 0.15, t = 0.865, *p* = 0.4011, df = 14.36). Claude showed similar patterns with gastroenterologists scoring 0.25 and dietitians 0.4 for misinformation (mean difference = 0.15, t = 0.785, *p* = 0.4442, df = 15.86). Gemini responses were rated 0.15 by gastroenterologists and 0.4 by dietitians for misinformation content (mean difference = 0.25, t = 1.368, *p* = 0.1932, df = 13.69).

In contrast, misinformation ratings showed no significant differences between expert groups across any AI model (ChatGPT: *p* = 0.40, Claude: *p* = 0.44, Gemini: *p* = 0.19). Mean misinformation scores remained low for both groups, ranging from 0.15–0.4 across all models and evaluator types.

Effect sizes were small for scientific accuracy (Cohen’s d = 0.226–0.309) and clarity (d = 0.179–0.304) differences, while misinformation showed medium to large effect sizes (d = 0.450–0.828) despite non-significant *p*-values.

**Figure 2:**
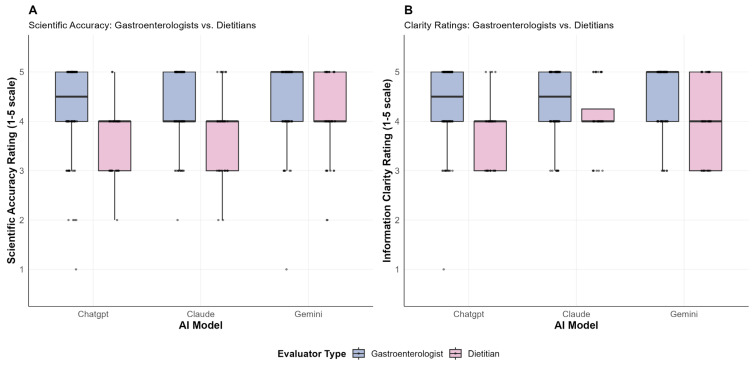
Evaluator type analysis for diet-related questions.

Figure 2 illustrates these findings.

### 3.6. Readability and Linguistic Analysis

Highly significant between-model differences were observed across all readability metrics with large effect sizes (all Friedman *p* < 0.001):

- Flesch Reading Ease: χ^2^ = 19.70, *p* < 0.001;
- Flesch-Kincaid Grade Level: χ^2^ = 22.55, *p* < 0.001;
- SMOG Index: χ^2^ = 23.97, *p* < 0.001.

Gemini 2.0 consistently demonstrated superior readability with the lowest educational requirements (Flesch-Kincaid Grade Level: median = 9.8, IQR = 8.8–10.3, equivalent to 9th–10th grade level), highest reading ease (Flesch Reading Ease: median = 48.8, IQR = 44.1–61.5, “fairly difficult” reading level), and lowest complexity (SMOG: median = 13.9, IQR = 13.1–14.4).

ChatGPT-4 required intermediate educational levels (Flesch-Kincaid Grade Level: median = 12.5, IQR = 11.6–15.3, college-level reading; Flesch Reading Ease: median = 38.2, IQR = 28.0–45.6, “difficult” reading level; SMOG: median = 14.9, IQR = 13.6–16.0), while Claude 3.7 presented the most linguistically complex responses (Flesch-Kincaid Grade Level: median = 12.5, IQR = 10.9–13.5; Flesch Reading Ease: median = 37.3, IQR = 30.9–42.3; SMOG: median = 16.0, IQR = 15.4–16.7, requiring college-level to graduate-level education).

**Figure 3:**
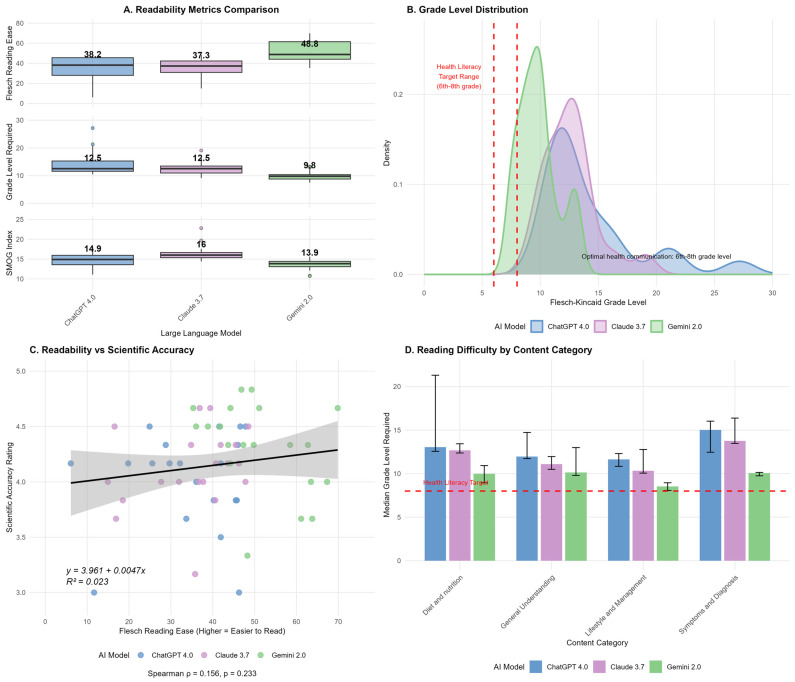
Readability analysis and scientific accuracy assessment of AI-generated health communication content across three large language models. This figure evaluates the readability and accessibility of health-related content generated by three AI models (ChatGPT-4, Claude 3.7, and Gemini 2.0) using multiple assessment frameworks. (**A**) Comparison of three readability metrics: Flesch Reading Ease scores (where higher values indicate easier reading), Flesch-Kincaid Grade Level, and SMOG index scores; (**B**) distribution of grade-level requirements across all generated content, with vertical dashed lines marking health literacy targets (6th–8th grade level recommended for health communication); (**C**) relationship between readability (Flesch Reading Ease) and scientific accuracy through a scatter plot; (**D**) reading difficulty by content category across different health topics, showing mean grade levels required for comprehension, with a horizontal dashed line indicating the optimal health literacy target.

Readability metrics were calculated on the original 60 AI-generated responses using validated computational linguistic measures. Significant between-model differences were observed across all readability metrics (all Friedman *p* < 0.001, large effect sizes: Flesch Reading Ease: Kendall’s W = 0.368, Flesch-Kincaid Grade Level W = 0.503, SMOG W = 0.589). These results are represented in Figure 3. Linear regression analysis examining the relationship between Flesch Reading Ease scores and scientific accuracy ratings revealed a weak, non-significant positive association (R^2^ = 0.023; Spearman ρ = 0.156, *p* = 0.233). This modest relationship suggests that readability and scientific accuracy represent largely independent quality dimensions, implying that achieving both high readability and high accuracy requires separate optimization strategies.

### 3.7. Performance Variation by Question Complexity

The data revealed significant variation in AI performance across individual questions, with certain queries consistently challenging all models while others demonstrated consistently high accuracy.

Highest-performing questions across all models included fundamental disease concepts. “What is celiac disease?” achieved mean scientific accuracy scores of 4.33 (ChatGPT-4), 4.33 (Claude 3.7), and 4.67 (Gemini 2.0). Similarly, “Is there a cure for celiac disease?” demonstrated strong performance with mean scores of 4.50, 4.67, and 4.33, respectively.

Lowest-performing questions consistently involved practical dietary management. “I have celiac disease. How can I prevent cross-contamination in my kitchen?” showed concerning accuracy across all models, with mean scientific accuracy scores of only 3.00 (ChatGPT-4), 3.17 (Claude 3.7), and 3.33 (Gemini 2.0). This question also demonstrated the highest misinformation detection rates, with 3, 5, and 5 expert identifications, respectively. ChatGPT-4′s response, while comprehensive, suggested “disposable products like paper towels, parchment paper, or even gloves while preparing gluten-free meals”. Claude 3.7′s response suggested color-coding as sufficient separation without adequately explaining why physical separation of equipment is necessary.

The question “What are the risks of not strictly following a gluten-free diet?” generated the most concerning misinformation. ChatGPT-4′s response (mean accuracy 3.00, 4 expert misinformation flags) understated the severity of long-term complications, particularly failing to adequately emphasize the nature of complications like intestinal lymphoma risk.

### 3.8. Follow-Up Question Generation

Analysis of AI-generated follow-up questions was conducted on the original AI responses. ChatGPT-4 generated follow-up questions in 85% of its responses (17/20 questions), Claude 3.7 in 70% (14/20 questions), while Gemini 2.0 rarely provided follow-up questions (20%, 4/20 questions). This pattern suggests different approaches to user engagement and information completeness across the model architectures.

### 3.9. Inter-Rater Reliability and Internal Consistency Analysis

Comprehensive ICC analysis using the ICC(2, k) model appropriate for fixed expert panels revealed variable reliability across models and metrics. For scientific accuracy, ChatGPT-4 demonstrated the highest inter-rater reliability (ICC_2,k_ = 0.51, 95% CI: 0.16–0.71), followed by Gemini 2.0 (ICC_2,k_ = 0.47, 95% CI: 0.10–0.71) and Claude 3.7 (ICC_2,k_ = 0.36, 95% CI: −0.14–0.66.

Clarity ratings demonstrated consistently poor inter-rater reliability across all models (all ICC_2,k_ < 0.25, all 95% CIs including negative values), indicating substantial subjective variability in clarity assessments.

Cronbach’s alpha analysis revealed acceptable internal consistency for scientific accuracy ratings within ChatGPT-4 (α = 0.645) and moderate consistency for Gemini 2.0 (α = 0.523) and Claude 3.7 (α = 0.465). Clarity ratings showed poor internal consistency across all models (ChatGPT-4 α = 0.306, Claude 3.7 α = 0.047, Gemini 2.0 α = 0.024).

## 4. Discussion

This comparative analysis of three leading LLMs reveals important differences in their performance for celiac disease patient education, with implications for clinical implementation and patient safety. Our evaluation demonstrates that while all models can generate medically sound content, significant variations exist in readability, misinformation prevalence, and content accessibility that directly impact their suitability for patient-facing applications.

The most striking finding was Gemini 2.0′s superior performance across multiple dimensions. Gemini 2.0 demonstrated statistically significant advantages in both scientific accuracy (*p* = 0.015) and clarity (*p* = 0.011), with significantly better readability requiring approximately 2–3 fewer years of education for comprehension (median Flesch-Kincaid Grade Level 9.8 vs. 12.5 for both competitors). Gemini also demonstrated lower misinformation rates (13.3%) compared to ChatGPT-4 (23.3%) and Claude 3.7 (24.2%), representing a clinically meaningful 40–45% reduction in potentially harmful content, although these differences did not reach statistical significance (*p* = 0.778). Gemini’s achievement of near-high school reading levels (9th–10th grade) brings AI-generated content closer to established health literacy recommendations, though all models still exceed the optimal 6th−8th grade target for patient materials.

Our findings align with previous research on LLM performance in healthcare applications. A study by Ayers et al. comparing physician and ChatGPT-4 responses to patient questions found that ChatGPT-4′s responses were preferred in 78.6% of cases and rated significantly higher for both quality and empathy, consistent with our findings of high clarity ratings across all models [85]. Our observation of generally high accuracy across all three LLMs (mean scores 4.02–4.42 out of 5) aligns with studies by Nori et al. demonstrating GPT-4 achieving passing scores on medical licensing examinations, [86] and Kung et al. showing ChatGPT-4′s capacity to perform at approximately the level of a successful medical student on USMLE-style questions [87]. A scoping review by AlSammarraie and Househ examining 69 comparative studies found ChatGPT-4 most frequently identified as the most accurate model [88]. The contrasting finding in the present study may reflect domain-specific variations in model performance, as supported by Aydin et al.’s scoping review of 201 studies [14], which found accuracy varied considerably by medical specialty, ranging from 46% excellent responses for pediatric in-toeing questions to 92.5% accuracy for hypertension queries.

Our work expands on previous research by directly comparing three leading models across multiple performance dimensions specifically for celiac disease education. A methodologically similar study by Song et al. compared multiple LLMs (Claude, GPT-4, BARD, and Bing) for urolithiasis information, finding that Claude demonstrated the highest accuracy [89]. Their contrasting finding suggests either domain-specific variations or significant improvements in Google’s models between generations.

The readability challenges we identified are consistent with findings from other medical specialties. Campbell et al.’s evaluation of ChatGPT-4 for obstructive sleep apnea patient education found mean Flesch-Kincaid grade levels exceeding 12th grade even with specific prompting for simpler language [90]. Similar patterns were documented by Cohen et al. for ophthalmology content [91]. Our finding that Gemini produces significantly more readable content (mean Flesch-Kincaid Grade Level 9.82, mean Flesch Reading Ease 51.19) adds a novel dimension to this literature. Chervonski et al. similarly found that Google’s Bard achieved better readability scores for vascular surgery information [92]. Aydin et al. noted that LLMs revealed a trade-off between comprehensiveness and readability [14], a pattern consistent across medical domains that underscores the challenge of achieving both informational depth and accessibility in AI-generated patient education materials. A novel finding emerged from our inter-professional evaluation framework: gastroenterologists consistently rated AI-generated content significantly higher than dietitians across all models for both accuracy and clarity, while both groups demonstrated equivalent sensitivity to misinformation detection. For scientific accuracy on diet and nutrition questions, gastroenterologists provided mean scores of 4.2–4.6 compared to 3.1–3.5 from dietitians across the three models (all *p* < 0.02). Similar patterns emerged for clarity ratings, with the largest disparity observed for Gemini (gastroenterologists: 4.8 vs. dietitians: 3.6, *p* = 0.0013).

These inter-professional differences carry substantial clinical implications. Dietitians’ more critical evaluation likely reflects their specialized training in translating nutritional science into practical, actionable guidance for patients. While gastroenterologists focus primarily on disease pathophysiology and medical management, dietitians routinely address the nuanced challenges of dietary implementation, including cross-contamination prevention, label reading, meal planning, and social dining situations. Their lower ratings suggest that AI-generated content, while medically accurate in broad terms, may lack the practical specificity and contextual awareness that dietitians recognize as essential for successful dietary adherence. These findings have important implications for AI validation processes and clinical practice. First, they underscore the critical importance of including registered dietitians in AI evaluation teams for conditions requiring dietary management, as their specialized expertise identifies practical limitations that medical specialists may overlook. Second, they suggest that current LLM training data may be weighted toward medical literature rather than practical dietary guidance, creating a gap between theoretical accuracy and real-world applicability. Third, they reinforce the established recommendation that celiac disease management requires multidisciplinary care teams, with dietitians playing an essential role that cannot be replaced by AI tools alone. Healthcare systems implementing AI educational tools should ensure dietitian involvement in content review and approval processes, particularly for diet-related queries where the gap between physician and dietitian assessments was most pronounced.

The misinformation prevalence we observed (16 expert identifications for Gemini 2.0 vs. 28–29 for other models) contrasts with findings by Almagazzachi et al. who reported 92.5% accuracy for ChatGPT’s responses to hypertension questions [93], and Caglar et al. who found 93–94% accuracy for ChatGPT in pediatric urology [94]. This suggests disease-specific variations in model performance. Zaretsky et al. reported that 18% of LLM-transformed discharge summaries raised safety concerns due to omissions or hallucinations [95], while McMahon et al. documented inaccurate information regarding self-managed medication abortion [96]. It is crucial to emphasize that there is no acceptable threshold for misinformation in patient-facing healthcare applications, and despite Gemini’s superior performance, the presence of any misinformation instances prevents recommending these models for direct patient use until misinformation rates reach zero.

The significant differences we observed in misinformation prevalence between models (16 expert identifications for Gemini 2.0 vs. 28–29 for other models) contrasts somewhat with findings by Almagazzachi et al. who reported 92.5% accuracy for ChatGPT’s responses to hypertension questions, suggesting potential disease-specific variations in model performance [93]. Similarly, Caglar et al. found high accuracy (93–94%) for ChatGPT in pediatric urology questions, with no completely incorrect responses [94]. Zaretsky et al. reported that while LLM-transformed discharge summaries showed improved readability and understandability, 18% of reviews identified safety concerns due to omissions or hallucinations [95]. McMahon et al. documented that ChatGPT provided inaccurate information regarding self-managed medication abortion, highlighting how misinformation can occur even on well-established clinical topics [96].

The lower performance on practical dietary management questions, particularly cross-contamination prevention (mean accuracy 3.00–3.33), warrants attention. Several factors likely contribute: practical recommendations require integration of multiple knowledge domains (food science, culinary practices, clinical guidelines); effective advice is context-dependent, varying by household setup and resources; rapidly evolving labeling regulations and product availability mean training data may not reflect current practices; and LLMs sometimes extrapolate from general food safety principles without considering celiac-specific thresholds (20 ppm). These findings suggest that LLM developers should prioritize specialized training data for lifestyle management content, including dietitian-reviewed protocols, and that healthcare providers should exercise particular vigilance when patients use AI for practical dietary advice.

Whether Gemini 2.0’s advantages justify clinical implementation depends on supervision level and acceptable risk thresholds. For unsupervised direct patient applications, current misinformation rates across all models exceed acceptable safety thresholds. For supervised applications, Gemini 2.0’s advantages become compelling in scenarios such as pre-visit preparation materials reviewed by providers, initial drafts for patient handouts requiring clinician verification, and post-consultation supplementary resources where clinical context has been established. Healthcare systems should establish mandatory clinician review protocols targeting higher-risk question types (practical dietary management, cross-contamination) and incorporate comprehension checks where providers ask patients about AI-derived understanding to identify misconceptions.

Several limitations should be considered. First, our study evaluated LLM performance at a single point in time, while these models undergo frequent updates. However, the core knowledge base for celiac disease management demonstrates relative stability, and our comparative framework offers a template for ongoing evaluation as new model versions emerge. Second, our evaluation relied on expert ratings, which inherently involve some subjectivity despite standardized assessment protocols. Third, our question set may not capture all patient information needs, and we did not evaluate varied prompting strategies. Additionally, our misinformation assessment employed binary detection without systematic categorization of error types; future studies should classify misinformation into categories such as factual errors, ambiguous expressions, and clinically significant omissions. While our questions underwent content validation, formal complexity-level testing was not performed prior to the study. Finally, we did not assess patient perceptions or comprehension of LLM-generated content, which would provide valuable insights into real-world utility.

These findings must be interpreted within the context of current patient behavior patterns. Rather than discouraging AI use, which patients are already employing, our findings suggest a path toward structured integration with appropriate clinical oversight. Gemini 2.0’s superior performance indicates that thoughtful model selection can significantly improve patient access to evidence-based information. Clinical workflows could incorporate AI-generated educational materials as starting points for patient discussions, with healthcare provider review ensuring accuracy and personalization. Future research should evaluate whether specific prompting strategies can improve readability and reduce misinformation rates, investigate patient comprehension and trust in LLM-generated materials, and extend similar comparative evaluations to other medical conditions to determine whether our findings are generalizable or disease-specific.

## 5. Conclusions

This study provides the first multi-expert comparative evaluation of leading LLMs for celiac disease patient education. Gemini 2.0 demonstrated significant advantages in accuracy, clarity, and readability over ChatGPT-4 and Claude 3.7; however, misinformation rates of 13.3–24.2% across all models preclude unsupervised patient applications. Future research should address multi-language evaluations, patient-centered outcomes, longitudinal performance tracking, and optimized prompting strategies to reduce misinformation to clinically acceptable levels.

## Figures and Tables

**Table 1:** Questions posed to LLMs.

Category	Question
General understanding	What is Celiac Disease?
	What causes celiac disease?
	Is celiac disease the same as gluten allergy or intolerance?
	Is celiac disease genetic? Can I pass it on to my children?
	Is there a cure for celiac disease?
Symptoms and diagnosis	What are the common symptoms of celiac disease?
	How is celiac disease diagnosed?
	Can I have celiac disease even if I don’t have digestive symptoms?
	Can I test for celiac disease if I’m already on a gluten-free diet?
	Can celiac disease develop later in life?
Diet and nutrition	What foods should I avoid with celiac disease?
	What are some safe, gluten-free alternatives to common foods?
	I have celiac disease. How can I prevent cross-contamination in my kitchen?
	Do I need to take supplements if I have Celiac disease?
	I have celiac disease. Can I ever eat gluten again?
Lifestyle and management	I have celiac disease. How long does it take for symptoms to improve after going gluten-free?
	How often should I follow up with my doctor after a celiac disease diagnosis?
	What blood tests are used to monitor celiac disease?
	I have celiac disease. Do I need a follow-up biopsy to confirm that my intestine is healing?
	What are the risks of not strictly following a gluten-free diet?

**Table 2:** Descriptive statistics for AI model performance.

	ChatGPT-4	Claude 3.7	Gemini 2.0	*p* Value
Scientific accuracy	4.0 [4.0–4.5]	4.0 [4.0–4.5]	4.5 [4.5–5.0]	0.015
Clarity of information	4.0 [4.0–4.5]	4.0 [4.0–4.5]	5.0 [4.5–5.0]	0.011
Misinformation rate (%)	23.3% (28/120)	24.2% (29/120)	13.3% (16/120)	0.778
Flesch Reading Ease	38.2 [28.0–45.6]	37.3 [30.9–42.3]	48.8 [44.1–61.5]	<0.001
Flesch-Kincaid Grade Level	12.5 [11.6–15.3]	12.5 [10.9–13.5]	9.8 [8.8–10.3]	<0.001
SMOG Index	14.9 [13.6–16.0]	16.0 [15.4–16.7]	13.9 [13.1–14.4]	<0.001

Values presented as median [IQR] for continuous variables, % (n/N) for categorical variables.

**Table 3:** Pairwise statistical comparisons between AI models.

Metric	Comparison	*p*-Value	Cohen’s *d*
Scientific Accuracy	ChatGPT-4 vs. Gemini 2.0	0.006	−0.70
	ChatGPT-4 vs. Claude 3.7	0.254	−0.24
	Claude 3.7 vs. Gemini 2.0	0.024	−0.52
Clarity	ChatGPT-4 vs. Gemini 2.0	0.002	−0.94
	ChatGPT-4 vs. Claude 3.7	0.022	−0.50
	Claude 3.7 vs. Gemini 2.0	0.053	−0.51
Misinformation	ChatGPT-4 vs. Gemini 2.0	0.074	0.45
	ChatGPT-4 vs. Claude 3.7	0.745	−0.08
	Claude 3.7 vs. Gemini 2.0	0.009	0.69

## Data Availability

The raw data supporting the conclusions of this article will be made available by the authors upon request.

## Abbreviations

The following abbreviations are used in this manuscript:

AI	Artificial Intelligence
GFD	Gluten-Free Diet
LLM	Large Language Model
SMOG	Simple Measure of Gobbledygook
